# Gummatous mitral valve endocarditis from tertiary syphilis

**DOI:** 10.1099/acmi.0.000817.v3

**Published:** 2025-05-08

**Authors:** Nisha George, Daniel Pan, Shirley Sze, Caroline Williams, Zein El-Dean, Victor Zlocha, Elizabeth Webb, Manish Pareek

**Affiliations:** 1 Department of Respiratory Sciences, University of Leicester, Leicester, UK; 2 Department of Infectious Diseases and HIV Medicine, University Hospitals of Leicester NHS Trust, Leicester, UK; 3 Leicester NIHR Biomedical Research Centre, Leicester, UK; 4 Li Ka Shing Institute of Health Information and Discovery, Oxford Big Data Institute, University of Oxford, Oxford, UK; 5 Development Centre for Population Health Sciences, University of Leicester, Leicester, UK; 6 WHO Collaborating Centre for Infectious Disease Epidemiology and Control, Li Ka Shing Faculty of Medicine, School of Public Health, University of Hong Kong, Hong Kong, PR China; 7 Department of Cardiovascular Sciences, University of Leicester, Leicester, UK; 8 Department of Microbiology, University Hospitals of Leicester NHS Trust, Leicester, UK; 9 Department of Cardiothoracic Surgery, University Hospitals of Leicester NHS Trust, Leicester, UK; 10 Department of Histopathology, University Hospitals of Leicester NHS Trust, Leicester, UK

**Keywords:** gumma, mitral endocarditis, syphilitic endocarditis, tertiary syphilis

## Abstract

A 50-year-old Romanian gentleman presented with fever, myalgia and 30 kg weight loss. He was treated for syphilis after acquiring it 16 years ago. On examination, there was a pansystolic murmur in the axilla, and the patient had an ataxic gait. Blood tests showed raised inflammatory markers. However, standard investigations for infective endocarditis, including multiple blood cultures, serological titres for fastidious organisms and antibody tests were negative. A computed tomography (CT) of the chest, abdomen and pelvis demonstrated hepatosplenomegaly with multiple splenic infarcts. A magnetic resonance imaging (MRI) of the head with contrast showed multiple punctate enhancement in the bilateral hemispheres with leptomeningeal enhancement. Transthoracic echocardiogram demonstrated a large vegetation leading to severe mitral regurgitation. Serum treponemal antibodies were positive; *Treponema pallidum* particle agglutination (TPPA) was positive at 1 : 1280, and rapid plasma reagin (RPR) 1 : 4 treponemal IgM was negative; lumbar puncture syphilis serology was negative. The patient was treated with an extensive period of intravenous antibiotics, in addition to a prosthetic metallic valve replacement, where unusual ragged calcified valvular tissue was observed. Tertiary syphilis is a difficult diagnosis to confirm, since it can often be indolent and occur in areas of the body where it may go unnoticed. In our case, a diagnosis of probable syphilitic endocarditis was made from a combination of the history, an initial increase in the size of the lesion following antibiotic therapy and observation of likely gumma on the mitral valve during surgery. In such cases, surgery in addition to optimal antimicrobial therapy is necessary for effective treatment. This case adds to the current literature that treatment with penicillin is likely inadequate to prevent late complications.

## Data Summary

All data are presented in the manuscript and in the supplementary video. The supplementary video can be found in Figshare, DOI: 10.6084/m9.figshare.27211806 [1].

## Case Presentation

A 50-year-old gentleman originally from Romania presented to our emergency department in the United Kingdom with a 4-week history of low-grade fever, generalized grade 1 myalgia, appetite loss and unintentional weight loss of 30 kg. He was previously treated for primary syphilis 16 years ago while imprisoned in Romania. He recalls that he was given a single intramuscular injection and was told treatment was completed. The man had multiple sexual partners in the last few months with occasional condom use. On examination, he was febrile at 38.2 °C, no palpable lymphadenopathy or rash and genitalia appeared normal with penile paraphernalia (Yakuza beads) but no ulceration or scars. All other examination findings were within normal limits. There was a grade 4 pansystolic murmur loudest at the apex radiating to the left axilla. The patient also had an ataxic gait, but other neurological examinations were negative. Blood tests showed raised inflammatory markers – C-reactive protein 100 mg l^−1^; White cell count 14.6x10⁹ l^−1^; neutrophil count 12.3×10⁹ l^−1^; lymphocytes 1.6×10⁹ l^−1^ – with normal haemoglobin, coagulation profile and liver function tests. Blood-borne virus screen (Human Immunodeficiency virus (HIV), Hepatitis B and Hepatitis C) was negative. Three blood cultures performed on separate occasions were negative for bacterial, mycobacteria or fungal organisms.

Infective endocarditis with neurological metastases was suspected. Computed tomography (CT) of the head with contrast showed focal regions of gyriform enhancement, with tiny, high-density foci. Chest, abdomen and pelvis CT demonstrated hepatosplenomegaly with multiple splenic infarcts. Subsequent magnetic resonance imaging of the head with contrast showed multiple punctate enhancement in the bilateral hemispheres with leptomeningeal enhancement. Transthoracic echocardiogram demonstrated large vegetation attached to the P3 scallop measuring 2x1.5 cm leading to severe mitral regurgitation.

Given these clinical findings, we empirically diagnosed tertiary syphilitic endocarditis but proceeded to rule out other causes. The patient was started on intravenous Ceftriaxone 2 g twice a day and oral Amoxicillin 2 g six times a day, which was initiated a week after admission. Serological testing for *Coxiella*, *Bartonella*, *Chlamydia, Legionella* and *Mycoplasma* was negative. Anti-streptolysin-O antibodies and autoimmune antibodies (Anti-nuclear antibody (ANA), anti-neutrophil cytoplasmic antibodies (ANCA), anti-phospholipid and anti-double stranded DNA) were also negative. Serum treponemal antibodies were positive; TPPA was positive at 1 : 1280, and RPR 1 : 4 treponemal IgM was negative. Lumbar puncture performed on the sixth day of antibiotic therapy showed a raised protein of 0.60 g l^−1^, normal glucose and raised white cells (26x 10^6 l^−1^) predominantly lymphocytes (82%); cerebrospinal fluid (CSF) culture, viral screen, meningococcal, pneumococcal and 16S PCR, however, were negative. CSF TPPA and RPR were also negative.

On day 31 of admission, the patient complained of dull, central chest pain for the first time since hospitalization. The pain was non-radiating and was not associated with diaphoresis or shortness of breath. The electrocardiogram (ECG) did not show any ST or T wave changes, nor any dynamic changes on repeat ECG. Troponin was mildly elevated which was static on the repeat troponin. A repeat trans-thoracic echocardiogram demonstrated an increase in the size of the vegetation, with the erosion of the mitral valve despite prolonged antibiotic therapy. The patient was switched from Amoxicillin to intravenous Teicoplanin 1 g once a day on day 38 of hospitalization with continuation of Ceftriaxone. A trans-oesophageal echocardiogram with concurrent angiogram showed a thickened calcified P3 scallop, severe mitral regurgitation, a functionally dilated left ventricle with a preserved ejection fraction of 54% and moderate stenosis of the right coronary with normal left branch (Video S1, available in the online Supplementary Material).

**Fig. 1.**
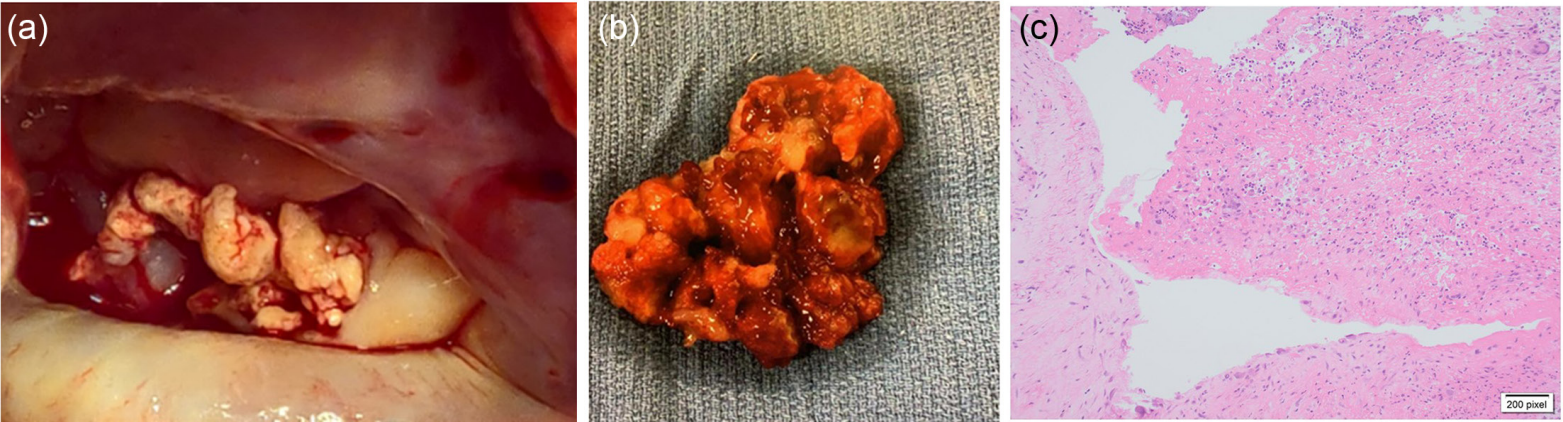
(a): Intra-operative photo of vegetation. (b) Macroscopic photo of excised vegetation. (c) Microscopic picture of vegetation

Due to the severity of the vegetation, the patient underwent a prosthetic metallic mitral valve replacement. Macroscopic pictures of the native valve demonstrated ragged calcified valvular tissue (Fig. 1a, b). Valve histology with the use of Warthin–Starry stain showed organizing vegetation with overlying fibrin and eosinophilic debris which was focally associated with neutrophils and were areas of granulation tissue (Fig. 1c). 16S, microscopy and culture of samples were negative. Following mitral valve repair, after a total of 61 days of Ceftriaxone, 43 days of Amoxicillin and 31 days of Teicoplanin, the patient was discharged from our care 53 days after his surgery on lifelong warfarin with no complications.

We present the first documented case of probable mitral valve syphilitic endocarditis within the literature. Tertiary syphilis is a difficult diagnosis to confirm, since it can often be indolent and occur in areas of the body where it may go unnoticed. The disconnect between the degree of elevation of TPPA (treponemal test) in contrast to RPR antibodies (non-treponemal test), in combination with the history, suggests that the patient had inadequate treatment for syphilis in the past, leading to late complications [2,3]. In our case, a diagnosis of syphilitic endocarditis was made from a combination of the history, an initial increase in the size of the lesion following antibiotic therapy (indicative of an immune reaction), and observation of likely gumma on the mitral valve during surgery in combination with likely gummatous lesions in the brain [4]. Microbiological yield from gumma is notoriously poor; however, in this case, our patient potentially fits the 2023 Duke-ISCVID clinical criteria for definite infective endocarditis (significant new valvular regurgitation on echocardiography/evidence of infective endocarditis documented by direct inspection during heart surgery, as well as fever, vascular phenomena from splenic abscesses), if positive syphilis titres are taken as part of the criteria, in the absence of any other positive microbiological data [4,6]. In such radical cases, surgery in addition to optimal antimicrobial therapy is necessary for effective treatment.

## Abbreviations

ANA: anti-nuclear antibody
ANCA: anti-neutrophil cytoplasmic antibodies
CSF: cerebrospinal fluid
CT: computed tomography
ECG: electrocardiogram
MRI: magnetic resonance imaging
RPR: rapid plasma reagin
TPPA: treponema pallidum particle agglutination
